# Supplementary material for: SCGAN-generated skin-tone-balanced extension of the HAM10000 dataset for fairer dermatological AI
Source: PLoS One. 2026 Apr 17;21(4):e0331081. doi: 10.1371/journal.pone.0331081 (PMC13089689; doi:10.1371/journal.pone.0331081)
Supplement: Attachment — Submitted filename: Response to Reviewers.pdf [file pone.0331081.s001.pdf]

# Response to Reviewers

I sincerely thank the Editor and both reviewers for their careful evaluation of our manuscript and for their constructive and insightful comments. Below, I provided a detailed point-by-point response to each comment. All changes have been incorporated into the revised manuscript.

---

## Reviewer #1

---

### Comment (1): Fairness claims lack quantitative validation

1. **Added a quantitative baseline classification experiment**:
   A simple ResNet-18 classifier was trained under two settings:
   (i) using HAM10000 only, and
   (ii) using HAM10000 augmented with the proposed synthetic dataset.
   We report accuracy and macro-averaged F1-score for both settings.

2. **Toned down fairness-related claims throughout the manuscript**:
   The language has been revised to clearly state that the dataset is intended as a controlled experimental resource for fairness-aware research, rather than claiming direct or guaranteed bias mitigation in clinical systems.

### Comment (2): Clarification of lesion-class conditioning

The generator is explicitly conditioned on skin tone only via a two-dimensional one-hot vector. Lesion class conditioning is not injected into the generator. Instead, lesion labels are assigned post-generation, proportionally to match the original HAM10000 class distribution. This design choice was made to preserve dataset-level class balance without enforcing class-specific generation constraints.

All statements implying explicit lesion-class conditioning within the generator have been corrected for accuracy.

### Comment (3): Justification of RGB tint blending and skin tone definition

In the revised manuscript, we explicitly state that the tone definitions used in this work are approximate and operational, rather than strict mappings to clinical scales such as Fitzpatrick or Monk.

The RGB tint vectors and blend strengths are presented as controlled visual proxies designed to introduce consistent, distinguishable tone variation in the absence of reliable skin tone annotations in HAM10000.

We now emphasize that these choices support controlled experimentation and fairness analysis, rather than clinical skin tone classification.

### Comment (4): Reproducibility details (batch size, hardware, etc.)

We have expanded the Methods section to include:

- Batch size
- Number of iterations per epoch
- Training duration
- Hardware configuration (single-GPU training environment)

Additionally, a code availability statement has been added to ensure full reproducibility, in line with PLOS ONE guidelines.

### Comment (5): Clarification of SCGAN vs existing GAN frameworks

In brief, SCGAN was chosen because it enables explicit attribute-controlled synthesis without requiring paired domain translation or well-defined source–target mappings, which are prerequisites for frameworks such as StarGAN or CycleGAN. SCGAN allows stable, large-scale generation of tone-balanced data even when real medium- and dark-tone reference domains are sparse or inconsistently labeled. We presented the reasons of choice in the revised manuscript.

## Reviewer #2

---

### Comment (1): Excessive implementation details in main text

Detailed folder structures and file-naming conventions have been removed from the main text and are now referenced implicitly via the dataset README hosted in the public repository. This improves readability and aligns the manuscript with scholarly presentation standards.

### Comment (2): Justification of GAN architecture choice

We have expanded the Methods and Discussion sections to provide clearer justification for the architectural design, including the choice of a tone-conditioned GAN, ResNet-based generator blocks, and PatchGAN-style discriminator. The discussion now explicitly links these design choices to the study's goals of controllable tone synthesis, lesion feature preservation, and computational efficiency.

### Comment (3): Lack of quantitative evaluation

We included the following:

1. Fréchet Inception Distance (FID) as a reference-level distribution similarity metric
2. A baseline classification experiment demonstrating the impact of synthetic augmentation

We clearly state the limitations of these metrics and frame them as **reference indicators**, not as measures of diagnostic realism.

### Comment (4): Absence of baseline downstream task evaluation

We agree and have added a baseline classification experiment, as described above. While intentionally simple, this experiment demonstrates that the synthetic dataset can positively influence downstream learning under controlled conditions.

---

## Closing Statement

I again thank the reviewers for their constructive feedback. I believe the revised version more accurately reflects the capabilities and limitations of the proposed dataset and will serve as a useful resource for fairness-aware dermatological AI research.

Sincerely,
**Safrina Kabir**
Sole Author and Corresponding Author
